# Predictor effect of Locus Of Control (LOC) on self-care activities and metabolic control in individuals with type 2 diabetes

**DOI:** 10.7717/peerj.2722

**Published:** 2016-11-23

**Authors:** Dilek Büyükkaya Besen, Neslihan Günüşen, Hamdiye Arda Sürücü, Cansu Koşar

**Affiliations:** 1 Faculty of Nursing, Dokuz Eylül University, İzmir, Turkey; 2 School of Nursing, Dicle (Tirgris) University, Diyarbakır, Turkey; 3 School of Nursing, Celal Bayar University, Manisa, Turkey

**Keywords:** Type 2 diabetes, Turkey, Self-care activities, Locus of control, A1c, Nursing

## Abstract

**Background:**

Previous studies have examined the role of individuals’ personal characteristics in diabetes management and used the locus of control theory to assess adherence to a diabetes management regimen. These studies have emphasized that having internal locus of control may be a protective factor in diabetes management.

**Objective:**

The purpose of this study is to determine the predictor effect of locus of control on self-care activities and A1c level.

**Method:**

The study is descriptive and relational. Researchers used a Diabetes Self-Care Activities Scale and a Locus of Control Scale to collect data. The study sample consisted of 129 individuals with type 2 diabetes.

**Results:**

The average score of locus of control of individuals with diabetes was 10.26, and the frequency of self-care activities in the past week was 2.9 days. A weak but statistically significant negative relation was found between the locus of control level and self-care activities of individuals with diabetes, which had no effect on A1c. It was determined that locus of control predicts 19% of self-care activities.

**Conclusion:**

According to the study results, having internal locus of control had positive effects on self-care activities. Training and planning activities to improve internal locus of control can improve diabetes management.

## Introduction

Diabetes mellitus has life-long acute and chronic complications, and it adds social and financial burdens on individuals, families, and society. The number of individuals with diabetes increases despite improvements in treatment. The World Health Organization stated that worldwide there are more than 346 million individuals with diabetes, and that this number will double by 2030 if no action is taken (WHO, 2015). The prevalence of diabetes in Turkey increased to 13.7% according to TURDEP II (2010) data, up from 7.2% according to TURDEP I (1997) data (Satman et al., 2010; Satman et al., 2002). Increase in life expectancy and especially changes in lifestyle (i.e., sedentary lifestyle and eating habits) have contributed to this rapid increase, both in the world and in our country (WHO, 2015; Satman et al., 2010; Wild et al., 2004; Satman et al., 2002).

Diabetes management is not easy for individuals. Diabetes may involve several physical and psychological problems, and successful management requires individuals to change their attitude and lifestyle, including exercise, blood sugar monitoring, and medication (Lutfey & Wishner, 1999). It is an essential problem that the majority of the diabetes complications are found in individuals who fail to make changes in their attitude, lifestyle, and monitoring of the disease. Research shows that 20% of individuals with type 2 diabetes do not monitor blood glucose levels (Evans et al., 1999) and only 30% of them have an exercise program (Kamiya et al., 1995). Most important, 53.6% of individuals with diabetes rely on physicians rather than themselves to manage and take responsibility for their own disease (Ghafoor & Riaz, 2011).

As with many other diseases, in order to manage diabetes and achieve the desired metabolic goals, the patient has to take a voluntary and active role in treatment. Individuals who believe that they control their health engage in more activities to reach that goal (Rodin, 1986).

Locus of control is defined in terms of whether individuals believe that outcomes of events are due to internal or external causes. Individuals with internal locus of control believe that they have control of the situations they face, and that they are responsible for managing their lives. Individuals with external locus of control believe what happens to them is beyond their control, and they attribute everything they face to fate, others, or chance (Rotter, 1966). According to Rotter (1966), “internal versus external control refers to the degree to which persons expect that a reinforcement or an outcome of their behavior is contingent on their own behavior or personal characteristics versus the degree to which persons expect that the reinforcement or outcome is a function of chance, luck, or fate, is under the control of powerful others, or is simply unpredictable.” LOC affects how one can cope with a stressful situation. Since those with internal LOC are proactive, they display more problem-focused coping skills than those with external LOC (Dağ, 2002). It is important for individuals to encounter a problem while dealing with day-to-day issues in their own areas of responsibility. If individuals think that they have no responsibility when a problem arises or is solved, then they do not attempt to solve the problem (Eskin, 2009). In terms of the improvement of internal locus of control, results of applications support the long lasting effectiveness of cognitive-restructuring technique that aims to change cognitive structure (Eskin, 2009).

Previous studies have examined the role of individuals’ personal characteristics in diabetes management. These studies have emphasized that having internal locus of control may be a protective factor in diabetes management. A study by O’Hea et al. (2005) found that diabetes of individuals with external locus of control progressed more severely and their A1c levels were higher than those of individuals with internal locus of control. A study by Morowatisharifabad et al. (2010) found a positive relation between internal locus of control and adaptation to diabetes treatment. Therefore, developing internal locus of control is a factor to consider in improving individuals’ adaptation to diabetes treatment (Montague, Nichols & Dutta, 2005).

A solid metabolic control of diabetes needs to be ensured because of its severe complications; therefore, factors that may have affect diabetes management need to be determined. Individual factors are important in ensuring the metabolic control of diabetes. Literature shows different research results on the effect of locus of control on metabolic control of diabetes and self-care activities. While some studies support a positive relation between internal locus of control and metabolic control of diabetes, in other studies, no relation was found (Hummer, Vannatta & Thompson, 2011; Bunting & Coates, 2000; Kneckt, Syrjala & Knuuttila, 1999). The findings of this study will contribute to the literature in explaining this effect and the results of this study can be used in planning diabetes education programs.

## Materials and Methods

### Study design

This study is a descriptive and relational study to determine the predictive effect of locus of control on self-care behavior and metabolic control in individuals with type 2 diabetes.

### Settings

Research data were collected from individuals diagnosed with diabetes who sought treatment at the diabetes polyclinics of two university hospitals between July 2013 and April 2014. The participants of the study consist of 129 individuals registered at Diabetes Education Centre of two hospitals who received either training with individual or group education programme. Licensed diabetes nurses were employed at both centres. Diabetes Education Centre received individuals with type 2 diabetes and they were expected to make recorded controls every three or six months. Each Centre also provided individuals with diabetes and group education as well as consultancy service. Education topics were such as living with diabetes, insulin therapy, healthy diet and exercise, and foot care, etc. Educators helped participants focus on the diabetes information most relevant to them and their experiences.

### Participants

The research sample included individuals over 18 years of age who had been diagnosed with type 2 diabetes for one year or longer.

### Inclusion criteria

• Individuals who had no disabilities related to seeing, hearing, or perception, or any other physical disability.
• Individuals who voluntarily agreed to participate in the study.
• Individuals whose A1c results of the past six months were available.

### Exclusion criteria

• Individuals with mental/cognitive problems.
• Individuals who were dependent on others for self-care activities due to an illness other than diabetes (cerebrovascular accident, chronic obstructive pulmonary disease, immobility, etc.).
• Individuals who were illiterate.
• Individuals who had disabilities related to vision, hearing, or perception, or any other physical disability.
• Individuals who did not voluntarily agree to participate in the study.

### Instruments

Data collection instruments to evaluate metabolic control were Identification Information Form, Diabetes Self-Care Activities measure (DSCA), Rotter’s Internal-External Locus of Control Scale, and A1c values in patients’ files.

### Identification information form

Introductory information forms were prepared by the researchers in line with the literature and were made up of 15 questions regarding socio-demographic characteristics and diabetes-related characteristics of the participants (years of diabetics, complication related to diabetes etc.) Socio-demographic information about gender, age, education level, marital status, employment and income level were obtained via self-report. Diabetes-related characteristics regarding the duration of the disease and diabetes education received were determined with self-report as well. Information about diabetes complications and non-diabetic chronic disease during the last six months was obtained from the patients’ medical records. The researcher searched these records for the reports of five types of complications related to diabetes: renal, ophthalmic, cardiovascular, neurological and of the peripheral vascular system. If the diabetes individuals suffered from at least one of these complications, they were then considered to have diabetes complications. Information about diabetes medication was obtained from patients’ medical records.

### Locus of control scale

Locus of Control Scale measures the position of individuals’ generalized control expectations and the belief that situations are controlled by either internal or external powers (chance or destiny) (Rotter, 1966). The scale consists of 29 items and two choices of phrases for each item, with a possible score between 0 and 23. Participants score one point for each of the following: 2.a, 3.b, 4.b, 5.b, 6.a, 7.a, 9.a, 10.b, 11.b, 12.b, 13.b, 15.b, 16.a, 17.a, 18.a, 20.a, 21.a, 22.b, 23.a, 25.a, 26.b, 28.b, and 29.a. A high score indicates external locus of control. In the adaptation of the scale in Turkey, Cronbach’s Alpha internal consistency coefficient was found to be .71, while test–retest reliability was found to be .83. Criterion-referenced validity of the same study was found to be *r* = .69 (*p* < .001) (Dağ, 1991). The scale was scored using a 5-point Likert scale: 1 = totally inappropriate, 2 = rather inappropriate, 3 = appropriate, 4 = quite appropriate, and 5 = totally appropriate. While the higher scores indicate tendency to external LOC, the lower scores indicate tendency to internal LOC. There are no cutoff points in the evaluation of the scale (Rotter, 1966).

### Diabetes self-care activities questionnaire

Diabetes Self-care Activities Questionnaire (DSCAQ) was developed by Toobert, Hampson & Glasgow (2000) to determine self-care activities of individuals with type 2 diabetes and adapted to Turkish by Coşansu & Erdoğan (2014). This scale allowed participants to report how well they were adhering to their specific regimen (Toobert, Hampson & Glasgow, 2000). The patients were asked how many days within the last seven days (day/week) they performed their self-care needs, which include diet, exercise, blood sugar testing, and foot care. The scale is composed of 11 items and five sub-dimensions, and these dimensions were diet (items 1, 2, 3, and 4); exercise (items 5 and 6); blood glucose monitoring (items 7 and 8); foot care (items 9 and 10) and smoking (Item 11). However, since it did not serve the purpose of the present study, the dimension of smoking was not used in the study. Cronbach alpha (*α* co-efficients) values of the sub-dimensions of the original DSCAQ were as follows: diet, .59; exercise, .70; blood glucose monitoring, .94; and foot care, .77 (Coşansu & Erdoğan, 2014). Each sub-dimension of the scale is scored separately and might be used independently. The score of each sub-dimension varies between 0 and 7. Total score of the scale being high indicates that self-care activities of the individuals with diabetes are good.

### A1c measures

For each individual with diabetes at the diabetes polyclinics, “Type 2 Diabetes Data Collection and Education Control Form” was filled and archived. When patients visited the diabetes polyclinics for routine follow, patient data updated. Patients values of A1c were taken from these records. A1c values of individuals covered by the study were examined in the Biochemistry Laboratory of the university hospital and data for study were obtained from the database of the hospital. A1c analysis was conducted in the laboratory of the hospital using an Adams A1c HA-8160 model Blood Analyzer.

### Data collection

Research data were collected from individuals diagnosed with diabetes who sought treatment at the diabetes polyclinics of two university hospitals between July 2013 and April 2014. Data were collected using a one-time face-to-face private interview with all of the participants.

### Data analysis

Data were analyzed using the Statistical Package for the Social Sciences (SPSS). The statistical analyses of data included an independent sample *t*-test, bivariate correlations, analysis of variance (ANOVA) with Tukey’s HSD post hoc test, and linear regression.

### Ethical considerations

Researchers obtained permission from the ethics committee and the hospital where the study was conducted. The approval reference/number “2013/34-10” from the Non-invasive research ethics board of Dokuz Eylul University. All the patients were given information about the study and its aims, and were told that participation was voluntary with no negative repercussions for nonparticipation. Contact information was provided along with the questionnaire. The patients’ oral and written informed consents were obtained.

## Results

A total of 129 individuals (57.4% female, 42.6% male) participated in the study. Among these individuals, 38.1% were primary school graduates, 91.5% were married, 30.2% were employed, and 69.8% had income equal to their expenses. Individuals’ average length of time since diabetes diagnosis was 6.48 ± 6.44 years. Average duration of oral anti-diabetic drug (OAD) use was 1.86 years and average duration of insulin use was 5.54 years. Average duration of combined use of OAD and insulin was 5.68 years. Average diabetes years of using OAD group was 2.18 years and average diabetes years of using insulin group was 8.13 years. Average diabetes years of using combined OAD and insulin group was 8.50 years (Table 1).

**Table 1:** Disease-related characteristics of individuals with type 2 diabetes (*n* = 129).

	Min–max	Mean ± SD
Year with Diabetes	1.00–35.00	6.48 ± 6.44
Duration of OAD use—year	1.00–5.00	1.8 6 ± 1.09
Duration of Insulin use—year	1.00–35.00	5.54 ± 5.92
Duration of OAD and Insulin use—year	1.00–20.00	5.68 ± 4.66
Diabetes years of using OAD group	1.00–6.00	2.18 ± 1.46
Diabetes years of using Insulin group	1.00–35.00	8.13 ± 7.20
Diabetes years of using OAD and Insulin group	1.00–21.00	8.50 ± 5.57

Score averages of locus of control were 10.26. Frequency of self-care activities in the past week averaged 2.9 days. The average of diet scores as one of the sub-dimensions of the self-criteria activities scale was 4.2 days. The exercise score average was 1.7 days. The blood sugar scores averaged 3.7 days. The foot care score average was 0.8 days. The average of A1c values of individuals was 8.64 ± 2.58 (Table 2).

**Table 2:** Score distribution of individuals with diabetes with respect to locus of control and self-care activities.

	Mean ± SD
Locus of control	10.26 ± 3.19
Total self-care activities	2.97 ± .83
Diet	4.22 ± .83
Exercise	1.74 ± 1.76
Blood sugar testing	3.77 ± 2.16
Foot care	.87 ± 1.42
HbA1c level	8.64 ± 2.58

Among the participants, insulin was the most commonly used medication for diabetes treatment; 55% (Table 3), and 53.5% of the individuals had other chronic diseases while 66.7% had no complications of diabetes (Table 3).

**Table 3:** Locus of control, self-care activity level and A1c level of individuals with diabetes according to socio-demographic and disease-related qualities.

	Locus of control level			Self-care activity level		A1c level	
	*n*	Mean ± SD		Mean ± SD		Mean ± SD	
**Age**							
19–35	10	11.80 ± 3.15		2.88 ± .87		8.64 ± 2.45	
36–49	30	11.63 ± 3.21		2.75 ± .69		7.68 ± 2.32	
50–65	67	9.68 ± 3.02		3.07 ± .89		8.68 ± 2.57	
Above 65 years	22	9.45 ± 3.09		2.97 ± .83		9.82 ± 2.66	
**Significance level**		4.074	**.008**	1.088	.357	3.073	**.030**
**Gender**							
Female	74	10.93 ± 3.28		2.97 ± . 89		8.46 ± 2.62	
Male	55	9.36 ± 2.86		2.97 ± .77		8.87 ± 2.53	
**Significance level**		2.830	**.005**	−.004	.997	−.872	.385
**Marital status**							
Married	118	10.18 ± 3.15		2.95 ± .82		8.63 ± 2.59	
Single	11	11.09 ± 3.72		3.10 ± .99		8.74 ± 2.65	
**Significance level**		−.896	.372	−.533	.595	−.140	.889
**Existence of chronic diseases**							
Yes	69	9.68 ± 3.10		3.05 ± . 83		9.24 ± 2.40	
No	60	10.93 ± 3.19		2.87 ± .83		7.95 ± 2.63	
**Significance level**		−2.253	**.026**	1.249	.214	2.909	**.004**
**Existence of complications in diabetes**							
Yes	43	9.58 ± 2.80		3.06 ± .80		9.52 ± 2.16	
No	86	10.60 ± 3.34		2.92 ± .85		8.19 ± 2.67	
**Significance level**		−1.726	.087	.904	.368	2.832	**.005**
**Family history of diabetes**							
Yes	50	10.36 ± 3.11		3.00 ± .73		9.28 ± 2.78	
No	79	10.20 ± 3.26		2.94 ± .90		8.23 ± 2.37	
**Significance level**		.271	.786	.380	.704	2.294	**.023**
**Employment status**							
Employed	39	10.51 ± 3.64		2.90 ± .76		8.23 ± 2.59	
Unemployed	90	10.15 ± 2.99		3.00 ± .87		8.81 ± 2.57	
**Significance level**		.581	.562	−.604	.547	−1.179	.241
**Income–expense level**							
Income is less than expenses	39	10.38 ± 3.04		2.97 ± .83		8.45 ± 2.00	
Equal income and expense	90	10.21 ± 3.27		2.97 ± .84		8.71 ± 2.80	
**Significance level**		.282	.778	−.011	.991	−.523	.602
**Education level**							
Primary school	49	9.83 ± 3.19		3.06 ± .72		9.05 ± 2.41	
Secondary school	35	9.97 ± 3.03		2.94 ± .79		8.61 ± 2.53	
High school	35	11.00 ± 2.97		2.89 ± 1.01		8.12 ± 2.82	
University	10	10.80 ± 4.39		2.89 ± .93		8.49 ± 2.71	
**Significance level**		1.103	.350	.325	.808	.897	.445
**Diabetes medicine in use**							
OAD	37	11.59 ± 3.40		2.46 ± .70		6.65 ± 1.18	
Insulin	72	9.52 ± 2.88		3.18 ± .82		9.40 ± 2.57	
OAD and insulin	20	10.45 ± 3.20		3.14 ± .76		9.55 ± 2.60	
**Significance level**		5.507	**.005**	10.877	**.000**	19.847 7	** .000**
**Number of trainings**							
1 training	45	10.86 ± 3.50		2.85 ± .75		8.07 ± 2.62	
2 trainings	34	9.70 ± 2.64		3.21 ± .74		9.56 ± 2.92	
3 trainings	29	9.80 ± 3.37		3.18 ± .98		9.74 ± 1.84	
**Significance level**		1.557	.216	2.357	.100	5.024	**.008**

### Locus of control

A significant difference was found between the locus of control levels of participants according to their age groups. The groups of participants over 65 years old has greater internal locus of control than the younger groups (Tukey HSD; p.034). Men were significantly more likely to have internal locus of control (*X* = 9.36) than women (*X* = 10.93) (*p*.005). The group with chronic diseases was more likely to have internal locus of control than those without chronic diseases (*X* = 9.68; *p*.026). There was also a significant difference between groups with respect to diabetes treatment method and locus of control. Further analysis (Tukey HSD; *p*.001) found a difference in locus of control level in the group using insulin (*X* = 9.52; *p*.005) and the group using OAD as well as the group using OAD and insulin. The study found that the existence of diabetes complications, family history of diabetes, employment condition, income status, education status, and training on diabetes management did not make a significant difference among groups with respect to locus of control (Table 3).

### Self-care activities

In this study, self-care activities of diabetic individuals in the past week were as follows: diet for 4.2 days, blood sugar test for 3.7 days, exercise for 1.7 days, and foot care for 0.8 days. No significant difference was found among the self-care activities of the participants with respect to age, gender, marital status, employment status, income, education, other chronic diseases, existence of diabetes complications, family history of diabetes, and training on diabetes management. On the other hand, self-care activities of the group aged 50–65 years were higher than the other groups. Men’s self-care activity levels were higher than women’s levels. The group with two or more trainings on diabetes management had higher self-care activity scores. According to the diabetes treatment method used, the self-care activity score of the group using OAD was significantly lower than the other groups’ score (Tukey HSD; *p*.000) (Table 3).

### A1c

Gender, marital status, employment status, income status, and education level of individuals participating in the study did not lead to significant changes in A1c levels of the groups. A1c average values in the 36–49-year-old age group were significantly lower than the other groups (Tukey HSD; p.043). A1c levels of the group with another chronic disease were significantly higher than the group without another chronic disease (*X* = 9.24; *p*.004). Participants with diabetes complications (*X* = 9.52) had significantly higher A1c values (*p*.005) than those without complications (*X* = 8.19). Participants with a family history of diabetes had significantly higher A1c values (*p* .023) than those without a family history of diabetes (*X* = 8.23). The group using OAD had significantly lower A1c values than other groups (*X* = 6.65; *p*.000). The significant difference in the A1c levels according to the number of trainings on diabetes management was due to the fact that the A1c levels of those with only one training were lower (Tukey HSD, *p*, 025) (Table 3).

There was a weak but statistically significant negative relation between the locus of control level and self-care activities of individuals with diabetes (*r* − .446; *p*.000). A negative, weak, and significant relation was found between the locus of control level and sub-dimensions of the self-care activities scale, namely diet (*r* − .35), exercise (*r* − .21), and blood sugar monitoring (*r* − .30). The relation with the sub-dimensions of foot care (*r* − .16) was negative, weak, and insignificant. A negative, very weak, and insignificant relation was found between the locus of control level of individuals and their A1c values. There was a weak, significantly negative relation between the locus of control level and diabetes years (*r* − .20). There was a weak and statistically significant positive relation between the diabetes years and sub-dimensions of the self-care activities scale, namely diet (*r*.21), blood sugar monitoring (*r*.23). There was a moderate and statistically significant positive relation between the diabetes years and A1c level (*r*.30). There was a strong and statistically significant positive relation between the diabetes years and number of training on diabetes management (Table 4).

**Table 4:** Relation between locus of control, self-care activities, A1c levels, diabetes years and number of training of individuals with diabetes.

	Self-care activity score		Diet		Exercise		Blood sugar testing		Foot care		A1c level		Diabetes year		Number of training	
	*r*	*p*	*r*	*p*	*r*	*p*	*r*	*p*	*r*	*p*	*r*	*p*	*r*	*p*	*r*	*p*
Locus of control level	−.44	**.000**	−.35	**.000**	−.21	**.015**	−.30	**.000**	−.16	.064	−.09	.277	−.20	**.023**	−.14	.141
Diabetes years	.11	.190	.21	**.016**	−.13	.133	.23	**.008**	−.09	.282	.30	**.000**	1		.56	**.000**

Locus of control was predictive on 19% of the self-care activity scores of individuals and had no effect on A1c (Table 5).

**Table 5:** Predictor effect of locus of control on self-care activities and metabolic control of individuals with type 2 diabetes.

Independent variant–dependent variant	Regression coefficient	St error	*t*	*p*
Locus of control–self-care activities	−.446	.754	−18.604	.000
Adjusted *R*^2^: .192 *F*: 31.459 *p*:.000				
Locus of xontrol-A1c	−.096	2.58	−1.091	*p*:.277
Adjusted *R*^2^: .009 *F*: 1.190 *p*:.277				

## Discussion

In this study, there was a negative, weak, and significant relation between locus of control level and self-care activities, and locus of control was predictive on 19% of the self-care activity points of individuals. No relation was found between the locus of control and A1c levels.

No study was found in the literature that examined the relation between locus of control and self-care activities of diabetic individuals. This study revealed that self-care activity scores of individuals with a greater tendency toward internal locus of control were higher. This result showed that individuals with internal locus of control took more responsibility and made a greater effort to manage their diabetes. Studies in the literature demonstrated that 20% of the individuals with type 2 diabetes did not monitor their blood glucose (Evans et al., 1999) and only 30% of them followed an exercise program (Kamiya et al., 1995). In addition, 53.6% of the individuals with diabetes believed their doctors should manage their illness, rather than taking responsibility for it themselves (Ghafoor & Riaz, 2011). The findings of this study are important, as they demonstrate that having internal locus of control is a significant and important predictor of the patient taking responsibility for his or her own diabetes management. In this study, the frequency of self-care activities of diabetic individuals in the past week was as follows: diet for 4.2 days, blood sugar test for 3.7 days, exercise for 1.7 days, and foot care for 0.8 days. These findings are supported by the report of Toobert, Hampson & Glasgow (2000), based on seven researchers using a self-care activities survey. This report also states that individuals with diabetes had higher self-care activities. The frequency of diet practice was 5.1 days per week, which is close to the averages of 5.2–6 days per week reported by Kim et al. (2004) and 5–5.6 days per week reported by French et al. (2008). The blood sugar test was the second most frequently reported self-care activity. This finding conforms to the results of Toobert, Hampson & Glasgow (2000), Kim et al. (2004), and Davis, Bruce & Davis (2006). Exercise-related self-care activities of the study group were rather low. The study of Tan & Magarey (2008) of 126 diabetic people in Malesia revealed that more than half of the individuals had a sedentary or slightly active life. Another study (Nelson, Reiber & Boyko, 2002) revealed that only 31% of the individuals with diabetes observed the recommended exercise program. In the present study, foot care activities involving review of feet and shoes were done around one day per week, and were the least frequently reported self-care activities. This finding is much lower than those in previous literature (Kim et al., 2004; Toobert, Hampson & Glasgow, 2000). A study by Bell et al. (2005) reported that 64% of the older individuals with diabetes observed and controlled their feet every day, while 35.6% of them controlled their shoes every day. In the study of Sarkar et al., the percentage of the individuals with diabetes who observe foot care advice was 63%. Contrary to these findings, the studies in Iran (Khamseh, Vatankhah & Baradaran, 2007) and Malesia (Rahimah & Elenser, 2008) revealed that diabetic individuals had insufficient knowledge and practice of foot care.

In contrast to the studies that support a significant relation between internal locus of control and metabolic control of diabetes (Morowatisharifabad et al., 2010; O’Hea et al., 2005; Surgenour et al., 2000; Stenstrom et al., 1998), other studies do not support this relation (Hayes et al., 2000). In a study by O’Hea et al. (2005), the A1c levels of the individuals with external locus of control were higher than those with internal locus of control. In a study by Morowatisharifabad et al. (2010), a positive relation was found between internal locus of control and adaptation to diabetes management. No relation was found between the locus of control and metabolic control of diabetes according to some studies (Nugent & Wallston, 2016; Hummer, Vannatta & Thompson, 2011; Bunting & Coates, 2000; Kneckt, Syrjala & Knuuttila, 1999). In a study by Kneckt et al., no relation was found between the locus of control and A1c level. A1c level is affected by many biological factors; it is not surprising that locus of control does not significantly relate to it (Surgenour et al., 2000; Kneckt, Syrjala & Knuuttila, 1999).

### Relation of socio-demographic and disease-related factors with locus of control, self-care activities, and A1c level

#### Locus of control

In contrast to previous studies, this study found that the group over 65 years old has greater internal locus of control than the younger groups. A study by Kneckt, Syrjala & Knuuttila (1999), found youth to be related to internal locus of control, while the study by Morowatisharifabad et al. (2010) revealed that external locus of control increased with age. In this study, men had significantly greater internal locus of control than women. Aalto, Uutela & Aro (1997) did not find any difference in locus of control by gender, but Buckelew et al. (1990) found that younger male patients reported a stronger internal locus of control than older male patients did. This study found that the group with chronic disease had greater internal locus of control than those without chronic disease, and the group using insulin had greater internal locus of control than other groups. Having another chronic disease and using insulin may have caused individuals to consider their condition more seriously and thus to have greater internal locus of control. The existence of diabetes complications, family history of diabetes, employment status, income, education level, and the number of trainings about diabetes did not make a significant difference among groups with respect to locus of control. This is similar to the findings of previous studies. A study by Kneckt, Syrjala & Knuuttila (1999), found a significant relation only with age among the demographic variants; however the study of Morowatisharifabad et al. (2010) and other studies found that internal locus of control increased with education level (Macrodimitris & Endler, 2001; Morowatisharifabad et al., 2010; Hayes et al., 2000; Surgenour et al., 2000). In our study, there was a weak, significantly negative relation between the locus of control level and diabetes years.

#### Self-care activities

The self-care activities of the group aged 50-65 years were higher than for the other groups. Previous studies showed that self-care activities increased with age (Jordan & Jordan, 2010; Xu et al., 2008; Whittemore, Melkus & Grey, 2005; Nelson, Reiber & Boyko, 2002; Toobert, Hampson & Glasgow, 2000). There was no significant difference among groups with respect to gender, marital status, employment status, income, and education level. In the study of McCollum et al. (2005), women’s self-care activity scores were lower than men’s scores, but in Lloyd et al.’s (1993), women’s self-care activity scores were higher than men’s scores. Previous studies stated that income was not related to self-care behavior (Sarkar, Fisher & Schillingrer, 2006; Karter et al., 2000). Existence of another chronic disease did not make a significant difference with respect to self-care activities, while the average self-care activity scores of those with a chronic disease were higher than the scores of those without a chronic disease. This finding is in contrast to the findings of McCollum et al. (2005) and Bayliss et al. (2003). Self-care activity scores of the group using OAD was lower than scores of the other groups. The study found that the existence of diabetes complications, family history of diabetes, employment status, income, and number of training on diabetes did not make a significant difference among groups with respect to self-care activities.

#### A1c level

No significant difference was found among the groups for A1c values with respect to gender, marital status, employment status, and income of the individuals participating in the study. The average A1c values of the group aged 36–49 years were significantly lower than the other groups. In the study of Sasi Sekhar et al. (2013), a relation was found between age and A1c. Jordon’s study (2010) Jordon found a weak correlation with age. In the study of Sasi Sekhar et al. (2013), gender made a significant difference. Weak glycemic control was more prevalent among women. The literature showed a mixed opinion on how gender determined glycemic control in type 2 diabetes. Some reports (Nielsen et al., 2006; Power & Snoek, 2001) showed a gender inequality, while others (Kobayashi et al., 2003; Jonsson et al., 2000) indicated no difference between males and females.

A1c levels of those with another chronic disease were significantly higher than A1c levels of those without another chronic disease. Participants with diabetes complications had significantly higher A1c values than those without complications. Those with a family history of diabetes had significantly higher A1c values than those without a family history of diabetes. These results were expected. The study also found that the group using OAD has significantly lower A1c values than the other groups. This result was due to the shorter duration of diabetes (1.86 years ± 1.09). Unexpectedly, the study found that groups who had two or more trainings on diabetes management had higher A1c levels. This result can be explained by the positive relation between diabetes years and A1c and the positive relation between diabetes years and number of trainings on diabetes management.

### Strengths and limitations of the study

This study’s main strength is that it is the first study to look at the relation between locus of control and self-care activities of individuals with diabetes. Therefore, the results are novel. Lack of literature created a limitation in explaining the relation between the locus of control, self-care activities, and demographic variants.

## Conclusion

This study found that having internal locus of control had positive effects on self-care activities. Diabetes management can be improved by trainings and planning activities about internal locus of control. Health professionals need to pay attention to trends of locus of control in individuals. There should be activities to improve internal locus of control.

##  Supplemental Information

10.7717/peerj.2722/supp-1 Data S1 Raw data exported from locus of control, self care activities, metabolic control and socio-demografic and diseases variable

## Additional Information and Declarations

### Data Availability

The following information was supplied regarding data availability: The raw data has been supplied as Data S1.
